# FIA-MS/MS-Based Targeted Metabolomics of Amino Acids and Acylcarnitines Uncovers Network-Level Metabolic Reprogramming in Chronic Kidney Disease

**DOI:** 10.3390/biomedicines14071622

**Published:** 2026-07-18

**Authors:** Luisa-Gabriela Bogos, Ioana-Ecaterina Pralea, Alin-Iulian Moldovan, Yuriy Maslyennikov, Andrada-Alina Bărar, Ștefan Ursu, Ina-Maria Kacso, Alina-Ramona Potra, Radu-Cristian Moldovan, Cristina-Adela Iuga

**Affiliations:** 1 Department of Personalized Medicine and Rare Diseases, MEDFUTURE Institute for Biomedical Research, Iuliu Hațieganu University of Medicine and Pharmacy Cluj-Napoca, 400012 Cluj-Napoca, Romania; 2 Drug Analysis Department, Faculty of Pharmacy, Iuliu Hațieganu University of Medicine and Pharmacy Cluj-Napoca, 400012 Cluj-Napoca, Romania; 3 Nephrology Department—Medical Specialties, Faculty of Medicine, Iuliu Hațieganu University of Medicine and Pharmacy Cluj-Napoca, 400012 Cluj-Napoca, Romania; 4 Department of Nephrology, Emergency County Hospital Cluj-Napoca, 400012 Cluj-Napoca, Romania; 5 Department of Surgery—Practical Skills, Iuliu Hațieganu University of Medicine and Pharmacy Cluj-Napoca, 400012 Cluj-Napoca, Romania; 6 Surgery Department, Prof. Dr. Octavian Fodor Regional Institute of Gastroenterology and Hepatology Cluj-Napoca, 400162 Cluj-Napoca, Romania

**Keywords:** chronic kidney disease, targeted metabolomics, flow-injection tandem mass spectrometry, amino acids, acylcarnitines, differential network enrichment analysis, energy metabolism, mitochondrial dysfunction, biomarker discovery

## Abstract

**Background/Objectives**: Chronic kidney disease (CKD) is a major global public health problem, and conventional biomarkers such as serum creatinine primarily reflect excretory function with limited sensitivity for early detection and for predicting progression. Amino acids (AAs) and acylcarnitines (ACs) reflect metabolic processes related to nitrogen metabolism and mitochondrial fatty-acid oxidation that are influenced by renal function. This study aimed to characterize CKD-associated alterations in AAs and ACs profiles using an integrated analytical framework combining differential analysis, supervised multivariate modeling, differential network enrichment analysis (DNEA), and cross-compartment plasma–urine profiling, moving beyond individual metabolite associations toward a multi-level characterization of CKD-associated metabolic reprogramming. **Methods**: Plasma and urine samples from 78 patients with CKD and plasma samples from 70 healthy controls were analyzed using flow-injection tandem mass spectrometry (FIA-MS/MS). An integrated targeted metabolomics framework combining differential, multivariate and network-based analyses including DNEA was applied to plasma and paired urine samples to characterize systemic and urinary metabolic alterations in CKD, focusing on AAs and ACs. **Results**: CKD was characterized by significant elevated short-chain dicarboxylic acylcarnitines, increased methylhistidine (MetHis) and argininosuccinic acid (ASA), together with reduced tryptophan, serine, methionine, and tyrosine in plasma. These metabolites were consistently identified across different analyses and correlated with kidney function markers. DNEA revealed coherent network-level reorganization, with acylcarnitine pathways gaining connectivity and centrality while amino acid modules lost integration in CKD. Cross-compartment analysis identified both systemic and compartment-specific patterns of metabolite distribution. An exploratory clustering-guided biomarker panel combining MetHis, C3DC, and Trp achieved an area under the curve (AUC) of 0.881 for discriminating patients with CKD from controls. Moreover, C6, C8, and C10 remained significantly associated with CKD after additional adjustment for estimated glomerular filtration rate (eGFR). **Conclusions**: Targeted metabolomic profiling revealed a coordinated metabolic signature in CKD suggesting disturbances in pathways related to fatty-acid oxidation, nitrogen imbalance, and altered amino acid metabolism. Network-level analysis provided evidence of systemic metabolic reorganization beyond individual metabolite changes. As findings derive from an observational cohort with high comorbidity prevalence, the identified signatures should be considered CKD-associated rather than CKD-specific.

## 1. Introduction

Chronic kidney disease (CKD) is a major global public health problem, affecting more than 788 million individuals (~14% of the adult population), with a steadily increasing incidence driven by aging, diabetes, and hypertension. Characterized by progressive and irreversible loss of kidney function, CKD is associated with high cardiovascular morbidity and mortality, and is projected to become one of the leading causes of death globally in the coming decades [1,2,3]. Current diagnosis and staging rely on the Kidney Disease: Improving Global Outcomes (KDIGO) criteria based primarily on eGFR and albuminuria [4]. Although widely used, traditional biomarkers, such as serum creatinine, reflect excretory function and provide limited information on underlying molecular mechanisms, early disease detection, or prediction of progression [5]. These limitations underscore the need for novel biomarkers capable of detecting early disease-related alterations, improving risk stratification, and supporting more personalized approaches to CKD management.

Beyond filtration and excretion, the kidney plays a central role in amino acid interconversion, nitrogen balance, and fatty-acid oxidation, and therefore acts as an important metabolic organ. Consequently, impairment of renal function leads to systemic metabolic disturbances reflected in both plasma composition and urinary excretion. Metabolomics enables the comprehensive profiling of small molecules and identification of pathway-level alterations associated with CKD development and progression [6,7,8]. Plasma and urine metabolite profiles provide complementary information: plasma reflects systemic accumulation and metabolic capacity, while urine provides direct information on tubular handling and renal clearance. Integrated analysis of both compartments may therefore help distinguish changes related to impaired renal excretion from those associated with broader metabolic dysregulation.

Previous metabolomic studies have reported disturbances in amino acid metabolism, mitochondrial energy metabolism and fatty-acid oxidation in individuals with CKD [9,10,11]. Among the most frequently reported alterations are changes in urea-cycle intermediates, tryptophan-derived metabolites, branched-chain amino acids and acylcarnitines suggesting extensive metabolic remodeling with declining renal function. Amino acid metabolism is particularly relevant to kidney dysfunction, with alterations in tryptophan, citrulline, glutamine, and branched-chain amino acids being associated with disease onset and progression [12]. Similarly, acylcarnitines reflect mitochondrial fatty-acid oxidation and energy metabolism, processes that are critically dependent on proximal tubular function, being disrupted early in CKD [13,14]. Together, these findings support the selection of amino acids and acylcarnitines as complementary targeted metabolite classes, capturing biologically relevant alterations that extend beyond conventional measures of kidney function, by reflecting key pathways involved in nitrogen metabolism, mitochondrial function, and energy homeostasis. Moreover, their simultaneous profiling enabling integrated assessment of interconnected metabolic pathways relevant to CKD. Targeted profiling of these metabolite classes is routinely performed using LC-MS/MS [15,16] and FIA-MS/MS, the latter offering significantly higher throughput [17].

Despite the growing body of metabolomics research in CKD, several important knowledge gaps remain. Most studies characterize metabolite alterations in plasma alone and report associations at the level of individual metabolites, providing limited insight into how metabolic relationships are reorganized during disease progression. Network-based approaches, such as differential network enrichment analysis (DNEA), can capture coordinated shifts in metabolite connectivity that may not be detected using conventional statistical analyses. Furthermore, the extent to which CKD-associated metabolic alterations are systemic or compartment-specific remains incompletely characterized, as integrated analyses of paired plasma and urine metabolomic profiles are rarely performed within the same cohort.

This study aimed to characterize plasma and urinary AA and AC profiles in CKD using targeted FIA-MS/MS within an integrated analytical framework. Specifically, we sought to identify CKD-associated metabolic alterations through covariate-adjusted univariate and multivariate analyses, evaluate network-level reorganization of metabolite associations using DNEA and characterize compartment-specific alterations through paired plasma–urine profiling. Moreover, a clustering-guided candidate biomarker panel was also proposed. Together, these approaches provide a multi-level characterization of CKD-associated metabolic reprogramming that extends beyond previously reported individual metabolite associations.

## 2. Materials and Methods

### 2.1. Sample Collection and Characteristics of the Patients

A total of 148 subjects were included in this study. The CKD cohort included 78 patients in various disease stages (prior to initiation of hemodialysis), enrolled prospectively, from whom paired plasma and urine samples were collected alongside relevant clinical characteristics. Considering that the prospective study design did not include the enrollment of non-CKD individuals, 70 plasma samples collected from individuals with no known kidney disease were included as a control (CTR) group; however, matching urine samples were not available for this cohort. For all subjects, venous blood was drawn through venipuncture in sodium heparin anticoagulant tubes after a minimum of 12 h fasting for each participant, while the urine samples were collected from the first urine in the morning. Both blood and urine samples were centrifuged at 1300 rcf for 10 min at 4 °C. Aliquots of plasma and urine were subsequently stored at −80 °C until analysis.

Demographic and clinicopathological data of the study participants are presented in Table 1 and Table S1. None of the participants in this study suffered from acute infections or cancer. Estimated GFR was calculated using the CKD-EPI equation, and CKD stage was defined according to KDIGO guidelines [4]. Hypertension stage was defined according to the European Society of Cardiology/European Society of Hypertension (ESC/ESH) guidelines [18] based on systolic and diastolic blood pressure measurements. Both CKD and CTR groups had comparable age and sex distribution.

### 2.2. Analysis of Amino Acids and Acylcarnitines

Amino acids and acylcarnitines were analyzed by FIA-MS/MS after derivatization with 10% acetyl chloride in 1-butanol (*v*/*v*), (Merck, Darmstadt, Germany). This well-established approach is routinely used for newborn screening of inborn errors of metabolism, but also applicable to other matrices [19]. In this study, plasma samples were processed as previously described [20,21], whereas urine samples were processed according to Kobayashi et al. [22] with minor modifications. In total, 112 metabolites including 21 AAs, 45 ACs, and 46 derived metabolic indicators were profiled (Table S2). Briefly, for plasma analysis, 5 µL of sample was extracted with methanol containing isotope-labeled AA and AC internal standards. The obtained extract was evaporated, derivatized at 65 °C for 20 min, and after a second evaporation step, reconstituted in 100 µL of mobile phase for FIA-MS/MS analysis. For urine analysis, 10 µL of sample were diluted 1:40 (*v*/*v*) with methanol, incubated for 30 min, and centrifuged for 5 min at 2500 rcf, after which 10 µL of supernatant was mixed with 100 µL internal standard, dried under nitrogen, derivatized at 65 °C for 20 min, and finally reconstituted in 100 µL of mobile phase prior to FIA-MS/MS analysis.

All analyses were performed on a Waters Acquity I-Class Plus liquid chromatograph coupled to a Waters Xevo TQ-XS mass spectrometer (Waters, Milford, MA, USA). At the beginning of each analytical batch, QC materials with assigned concentrations were analyzed as an independent check of instrument performance and analytical system suitability (Chromsystems, Gräfelfing, Germany). Plasma and urine samples were analyzed in randomized order, but in separate batches. Within-batch analytical repeatability and signal stability was assessed by injecting pooled plasma (or urine) QC samples after every eight study samples. These were prepared by combining equal volumes from each sample of plasma and urine, respectively. MS/MS detection was performed using different acquisition modes according to the analyte class (Table S3). Multiple reaction monitoring (MRM) was used for Gly, Leu, Orn, Lys, Met, Arg, Cit, argininosuccinic acid, and their corresponding internal standards; a neutral-loss scan of 102 Da was used for the remaining amino acids, whereas a precursor-ion scan of *m*/*z* 85 was used for free carnitine and acylcarnitines. Metabolites quantification was performed relative to stable isotope-labeled amino acids and acylcarnitines.

Primary data processing was performed using the MassLynx IonLynx module (v4.2, Waters, Milford, MA, USA). The resulting plasma metabolite concentrations, along with derived sums and ratios, are reported in Table S4. Urinary metabolite data were normalized to creatinine (expressed as metabolite-to-creatinine ratios) and are presented in Table S5. For the cross-compartment distribution analysis described in Section 3.4.4, normalized urinary concentrations were divided by corresponding plasma concentrations, allowing estimation of metabolite clearance.

### 2.3. Statistical Analysis

#### 2.3.1. Correlations and Network Analysis

Kendall’s tau (τ) correlations were calculated using SPSS Statistics (version 26; IBM Corp., Armonk, NY, USA) using a two-tailed significance threshold of *p* ≤ 0.05 (Table S6) and an effect size cutoff of |τ| > 0.26. Correlation networks were generated using Metscape (version 3.1.3 Cytoscape app.) and visualized in Cytoscape (version 3.10.3; Java 17.0.0) with node-level topological parameters calculated using the built-in Cytoscape Analyze Network function (Table S7). Urinary metabolites concentrations were normalized to urine creatinine prior to statistical analysis. To avoid circular associations, urine creatinine was excluded from correlation analyses involving urine data.

#### 2.3.2. Univariate and Multivariate Analysis

Metabolomics data were analyzed using MetaboAnalyst 6.0 (accessed in February 2026). Raw metabolite concentrations were log10-transformed and autoscaled (mean-centered and divided by standard deviation) prior to statistical analysis.

Potential confounding by age and sex was assessed using principal component analysis (PCA), correlation heatmaps, and covariate-adjusted linear models. Differential metabolite analysis between CKD and CTR groups was performed using linear models implemented in the limma framework (Linear Models for Microarray Data), with disease group as the main factor and age and sex (and eGFR) included as covariates (CTR as reference). *p*-values were adjusted for multiple testing using the Benjamini–Hochberg false discovery rate (FDR), with FDR-adjusted *p*-values < 0.05 considered statistically significant.

Multivariate analysis was performed to evaluate global metabolic differences and identify discriminative features, including partial least squares discriminant analysis (PLS-DA). Model performance was evaluated using cross-validation, and predictive ability was assessed using the Q^2^ statistic. Statistical significance of the model was determined using permutation testing (2000 permutations). Variable importance in projection (VIP) scores were calculated, and metabolites with VIP > 1 were considered important contributors to group discrimination. Data visualization (PCA, heatmaps, volcano plots, PLS-DA, ROC curves) was performed in MetaboAnalyst.

#### 2.3.3. Biomarker Analysis Methodology

Biomarker performance was evaluated using the ROC analysis module of MetaboAnalyst 6.0. Univariate ROC analysis was performed using 100-fold cross-validation and AUC, 95% confidence intervals (bootstrap), sensitivity, and specificity were calculated. A multivariable biomarker panel was constructed based on the following criteria: i. Significance in differential analysis (limma); ii. High variable importance in projection (VIP) > 1.0 (PLS-DA); iii. High univariate AUC (>0.70), and iv. Represented distinct K-means clusters (k = 5) to reduce redundancy. Logistic regression was used for panel construction, with performance assessed by repeated cross-validation and permutation testing (*n* = 1000).

#### 2.3.4. Differential Network Analysis

Differential network analysis was performed with the DNEA R package (v1.0.0; Patsalis et al. [23]) which jointly estimates condition-specific metabolite association networks under a Gaussian graphical model (GGM) framework. Because both groups are estimated in a single model, all 148 samples (78 CKD, 70 CTR) contribute to one sparse precision-matrix estimation by graphical LASSO over the 66 metabolites, each retained as an individual node with no feature aggregation. Joint estimation is appropriate for the *p* > *n* regime of these data, and the resulting partial correlations remove indirect (confounded) associations so that retained edges approximate direct metabolite–metabolite relationships. The L1 penalty was set with the asymptotic scaling rule λ = √(log(m)/n) (the package default), giving λ = 0.245; this data-adaptive value scales with dimensionality and sample size and is a principled, computationally efficient alternative to cross-validation, which is unstable when *p* > *n*. Edge robustness was ensured by stability selection over 1000 subsampling iterations, retaining only consistently selected edges and controlling the expected number of false positives; this yielded a network of 51 stable edges. Stable metabolic subnetworks were then identified with the built-in consensus clustering, which aggregates seven clustering algorithms and assigns a metabolite to a subnetwork only when at least 50% of them agree on its membership (agreement threshold τ = 0.5, i.e., ≥4 of 7 algorithms); this resolved six stable subnetworks (44 metabolites), leaving 22 metabolites unclustered. Finally, differential enrichment of each subnetwork was tested with NetGSA, which integrates node-level (abundance) and edge-level (connectivity) effects between the CKD and CTR groups; of the six subnetworks, the four sufficiently connected for testing were all significantly perturbed after Benjamini–Hochberg FDR correction (q < 0.05).

## 3. Results

### 3.1. Network Analysis of Clinical and Metabolomic Interactions

Correlation analysis identified a modular clinical network with three dominant domains: kidney function, mineral–bone metabolism, and hematological parameters (Figure S1A, Table S6). Network topology analysis further highlighted renal function-related variables including CKD stage, eGFR, serum creatinine, and serum urea, as central hubs with the highest degree and clustering coefficients (Table S7). As expected, CKD stage correlated strongly and positively with serum creatinine and urea and very strongly and inversely with eGFR, while creatinine and urea were strongly correlated with each other, associations consistent with progressive renal impairment. Markers of mineral–bone metabolism including phosphate, calcium, and parathyroid hormone (PTH), clustered closely with kidney function variables. Phosphate and PTH clustered with renal function markers and showed moderate-to-strong positive associations with CKD severity, whereas calcium was inversely associated with CKD stage, creatinine, phosphate, and PTH. Phosphate exhibited high betweenness centrality, linking renal and hematological parameters. Hemoglobin and hematocrit formed a tightly connected subnetwork and were strongly and negatively associated with CKD severity. In contrast, metabolic, inflammatory, and cardiovascular variables (glucose, lipids, C-reactive protein (CRP), blood pressure, body mass index) displayed predominantly weak-to-moderate correlations and remained peripheral. Notably, no hubs were identified outside the renal, mineral–bone, and hematological domains.

Within the plasma clinical–metabolite correlation network (Figure S1B), a distinct CKD-related metabolic core was identified based on moderate to strong associations with all four kidney function markers (CKD stage, eGFR, serum creatinine, and serum urea). This core included Cit, Trp, ASA, MetHis, and several dicarboxylic and hydroxylated acylcarnitines (C3DC, C4DC, C5DC, C6DC, C8DC, C5:1, and C5-OH). Interestingly, Glu correlated with CKD stage, eGFR, and creatinine but not urea. Asp was specific to CKD stage and C4-acylcarnitine was uniquely shared with serum urea. Mineral–bone parameters showed limited overlap, with ASA linked to phosphate, PTH, and calcium, MetHis to phosphate and PTH, and C4DC uniquely to PTH. Hematological variables displayed the broadest connectivity, sharing amino acids and predominantly dicarboxylic acylcarnitines, together with long-chain species. Seven metabolites were common to renal, mineral–bone, and hematological modules—namely, ASA, MetHis, Glu, C5:1, C5-OH, C4DC, and C8DC. Most CKD-core metabolites were positively associated with CKD severity markers and inversely with eGFR and hematological indices, whereas Trp showed the opposite pattern but retained high centrality. The highest node degrees were observed for dicarboxylic and hydroxylated acylcarnitines (C4DC, C8DC, C5:1, and C5-OH), followed by C5DC and C6DC, as well as ASA and MetHis, while very long-chain acylcarnitines (C24&C18:1DC and C26) displayed restricted hematological connectivity (Table S7).

In the urine clinical–metabolite network, kidney function markers formed the dominant axis (Figure S1C). Eight metabolites defined the urinary CKD core (C4DC, C5-OH, C5, C6, C10, Val, Glu, Cit), showing inverse correlations with CKD stage, creatinine, and urea and positive correlations with eGFR, except for Glu and Cit. C5:1, Lys, and Pro were moderately strong associated with filtration markers but not urea, indicating primary dependence on glomerular and tubular handling. Selective associations were observed for individual metabolites, with C10:1 linked to both eGFR and urea, His and Ser specific to urea, C12:1 to CKD stage, and C10:2 to serum creatinine. Mineral–bone associations were selective, with Cit shared across phosphate, PTH, and calcium, while hematological overlap was limited to Glu, Cit, C5-OH, and C10:1. Five metabolites (Cit, C4DC, C5-OH, Glu, Val) were common to all clinical modules. In contrast, several metabolites were exclusively associated with kidney function, including C5:1, C5, C6, C10:2, His, Lys, and Ser, highlighting urinary alterations in urinary amino acid and acylcarnitine handling. Network topology identified C4DC, C5-OH, Cit, Glu, and Val as the most connected urinary nodes, with higher centrality for short-chain dicarboxylic acylcarnitines, while long-chain species and several amino acids (e.g., His, Ser, Trp) remained peripheral (Table S7).

Comparison of kidney function-associated metabolites between plasma and urine identified a small, shared core (Glu, Cit, C4DC, C5:1, C5-OH) together with pronounced matrix-specific patterns. Urine associations were enriched in amino acids and short-to medium-chain acylcarnitines (Val, Pro, Lys, His, Ser, C5 to C12), whereas plasma showed additional links with ASA, MetHis, Trp, Asp, and multiple dicarboxylic acylcarnitines (C3DC, C4, C5DC, C6DC, C8DC). Mineral–bone metabolism showed limited overlap across matrices, with Glu, C5-OH, and C4DC shared. Plasma exhibited specific associations (ASA, MetHis, C5:1, C8DC), while urine was characterized by Cit, Pro, and Val. Hematological-related metabolites also displayed a small shared core (Glu, Cit, C5-OH, C4DC, Val) with concordant directions of association (Glu and Cit negative; C5-OH, C4DC and Val positive). Plasma showed a broader hematological profile, including amino acids (Trp, Ala, ASA, MetHis) and multiple dicarboxylic (C3DC, C5DC, C5:1, C6DC, C8DC, C20DC) and very long-chain acylcarnitines (C24&C18:1DC and C26), whereas urine associations were limited to three medium-chain species (C10, C10:1, C12:1).

### 3.2. Differential Analysis

As an initial step, exploratory analyses were performed to evaluate potential confounding effects of demographic variables and to characterize the overall structure of the plasma metabolomics data. Metadata correlation analysis showed no strong associations among age, sex, and group classification, indicating minimal collinearity and low risk of confounding (Figure S2A). PCA demonstrated partial separation between CKD and CTR samples along PC1 and PC2, which together explained 41.8% of the total variance, with no sex-driven clustering (Figure S2B). Hierarchical clustering showed a tendency for samples to cluster by disease status rather than demographic variables (Figure S2C).

#### 3.2.1. Univariate Analysis

**Figure 1:**
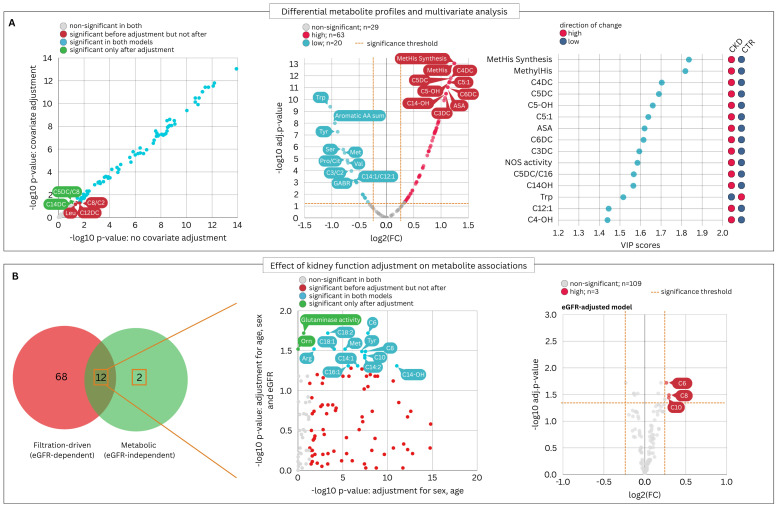
Differential and multivariate analysis of metabolomic profiles distinguishing CKD and CTR groups. (**A**) Differential metabolite analysis and multivariate modeling. Left: comparison of −log10-transformed *p*-values obtained from linear models before and after adjustment for age and sex. Middle: volcano plot showing differential metabolite abundance between CKD and CTR groups based on the age- and sex-adjusted model. Right: VIP scores derived from PLS-DA, ranking metabolites according to their contribution to group separation. The two columns to the right, labelled CKD and CTR, indicate the direction of change of each metabolite in the respective group (red circle, higher; blue circle, lower); (**B**) Effect of kidney function adjustment. Left: Venn diagram illustrating overlap between metabolites significant in the age- and sex-adjusted model and those remaining significant after additional adjustment for eGFR. Middle: comparison of statistical significance before and after eGFR adjustment. Right: volcano plot of the eGFR-adjusted model. In the volcano plots (panels (**A**), middle and (**B**), right), vertical dashed lines indicate the fold-change threshold (|log_2_FC| = 0.26) and the horizontal dashed line the significance threshold (adjusted *p* = 0.05); metabolites exceeding both are colored (red, increased; teal, decreased).

Comparison of linear models with and without adjustment for age and sex showed strong concordance, indicating minimal confounding. Only a small subset of metabolites changed significance status after adjustment. Specifically, C5DC/C8 metabolism indicator and C14DC became statistically significant, whereas C12DC, and C8/C2 were significant only in the unadjusted model (Figure 1A; Table S8).

The top 10 upregulated and 10 downregulated metabolites and metabolic indicators showing significant differential abundance between CKD and CTR groups are presented in Figure 1A (middle panel). CKD was characterized by higher levels of short-chain dicarboxylic acylcarnitines (C3DC, C4DC, C5DC, C6DC) and the amino acid-related metabolites MetHis and ASA. In contrast, several amino acids, including Trp, Tyr, Ser, Met, and Val were significantly reduced in CKD, with similar trend observed across related metabolic indicators.

To further distinguish between metabolites associated with impaired renal clearance and those reflecting intrinsic metabolic alterations, univariate analysis was repeated with additional adjustment for eGFR (Figure 1B). Adjustment for eGFR substantially changed the profile of metabolites associated with CKD. A total of 68 metabolites had strong dependence on kidney function being significant only in the age- and sex-adjusted model, but not after inclusion of eGFR. These metabolites were predominantly composed of short-chain, dicarboxylic, and hydroxylated acylcarnitines (e.g., C3DC, C4DC, C5DC, C6DC), as well as MetHis and ASA. In contrast, 12 metabolites presented eGFR-independent metabolic alterations including several amino acids (Tyr, Met, Arg) and selected long-chain acylcarnitines (e.g., C16:1, C18:1, C18:2). Additionally, Orn and Glutaminase activity were found significant only after eGFR adjustment. Importantly, a limited subset of metabolites (C6, C8, and C10) were both statistically robust in differentiating CKD and CTR and independent of renal function after applying an additional fold-change threshold.

#### 3.2.2. Multivariate Analysis

Supervised multivariate analysis was performed using PLS-DA. The model demonstrated separation between CKD and CTR groups, with the first two components accounting for approximately 40% of the total variance. Model performance was assessed using cross-validation, yielding a Q^2^ value of 0.36162, indicating moderate predictive ability. Permutation testing (2000 permutations) confirmed the statistical robustness of the model (*p* < 5 × 10^−4^) (Figure S3). Variables contributing most strongly to group separation were identified based on VIP scores derived from component 1. The highest-ranking features included MetHis Synthesis, MetHis, C4DC, C5DC, C5-OH, C5:1, ASA, C6DC, C3DC, NOS activity, C5DC/C16, C14-OH and Trp (VIP > 1.5) (Figure 1A—right panel). The full list of VIP scores is provided in Table S9. Notably, several of these metabolites overlapped with those identified as eGFR-dependent in univariate analysis. Based on this convergence, these metabolites were prioritized for subsequent biomarker evaluation.

### 3.3. Biomarker Analysis

**Figure 2:**
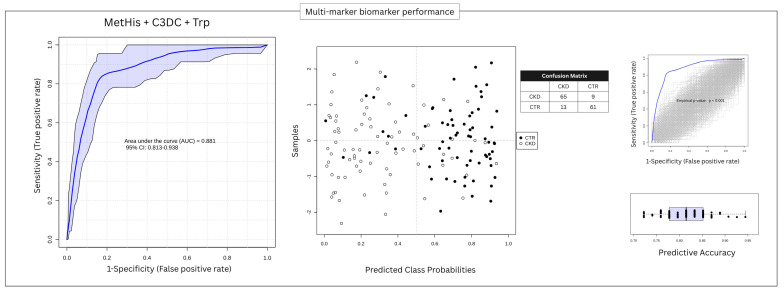
Performance of the three-metabolite biomarker model (MetHis, C3DC and Trp), including the predicted class probabilities, confusion matrix, and permutation-based model validation.

ROC analysis identified several metabolites with strong discriminatory performance between CKD and CTR groups (Table S10). The highest AUC values were observed for metabolites within Cluster 4, including MetHis and MetHis Synthesis (AUC = 0.88), ASA (AUC = 0.86), and short-chain dicarboxylic acylcarnitines such as C4DC and C5DC (AUC > 0.85). Acylcarnitines from Cluster 5 also showed high classification performance (AUC 0.80–0.84), whereas the amino acid-enriched Cluster 3 demonstrated moderate discrimination (AUC 0.73–0.83). In contrast, long-chain acylcarnitines and amino acid-derived indicators in Clusters 1–2 showed lower AUC values (0.50–0.66) (Figure S4). A multi-marker panel was constructed using representative metabolites from distinct clusters (MetHis, C3DC, and Trp) to reduce redundancy and capture complementary metabolic information. The three-metabolite model achieved robust discrimination (AUC = 0.881, 95% CI 0.813–0.938) (Figure 2) and improved classification compared with individual markers. Specifically, overall accuracy increased from 0.818 to 0.851, sensitivity from 0.779 to 0.824, and specificity from 0.859 to 0.878. The combined model reduced false negatives (17 to 13) and false positives (10 to 9), with cross-validated accuracy reaching 0.85. It should be noted that two panel members, MetHis and C3DC, are among the metabolites attenuated after eGFR adjustment (Section 3.2.1), whereas Trp retained eGFR-independent significance. The panel therefore represents a CKD-associated metabolic signature that partly reflects filtration-dependent burden—an intrinsic feature of CKD biology—rather than a signature independent of renal function.

### 3.4. DNEA of Plasma Metabolites in CKD Versus CTR

#### 3.4.1. Subnetwork-Level Organization

**Figure 3:**
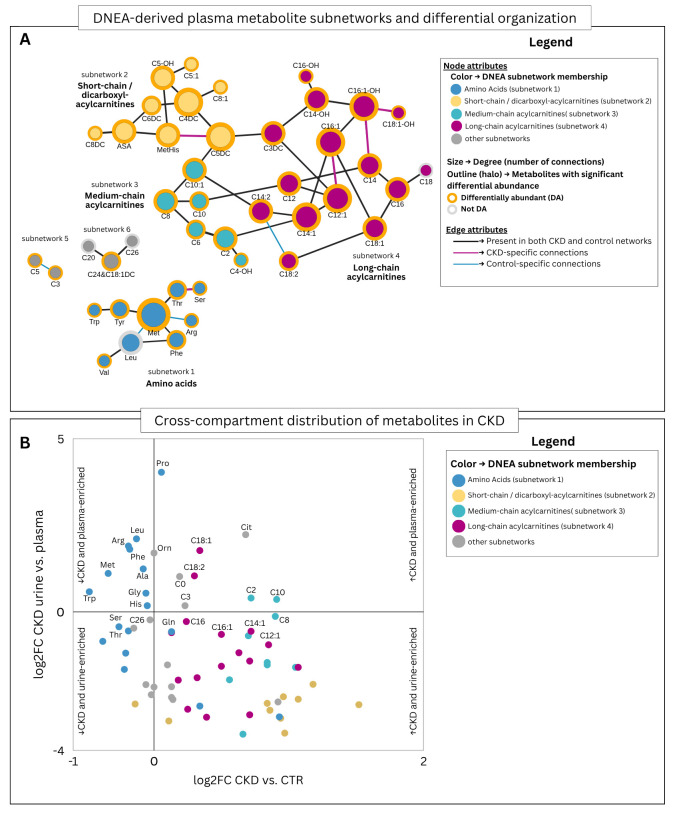
Network organization and cross-compartment redistribution of metabolites in CKD. (**A**) Differential plasma metabolites network identified by DNEA. Nodes represent metabolites and are colored according to subnetwork membership. Node size is proportional to degree (number of connections) and node outline indicates differential expression status. Edges represent significant partial correlations, with black edges shared between conditions, magenta edges specific to CKD, and blue edges specific to control. (**B**) Cross-compartment distribution of metabolites based on plasma log_2_ fold change (CKD vs. CTR; *x*-axis) and log_2_-transformed urine-to-plasma ratios (*y*-axis) derived from paired samples. Positive values indicate plasma enrichment, whereas negative values indicate urine enrichment. Metabolites are color-coded according to DNEA-derived subnetwork membership.

DNEA identified six subnetworks, along with a set of independent nodes, in the comparison between CKD and CTR plasma samples (Figure 3A, Table S11). The identified subnetworks varied in size, comprising between 2 and 15 nodes and 1 to 19 edges. Subsequent NetGSA revealed that four of the six subnetworks were statistically significant after FDR correction. Notably, these significant subnetworks corresponded to those exhibiting the highest proportion of differentially expressed (DE) nodes. Subnetwork 1 (9 nodes, 9 edges) comprised primarily amino acids, including Leu, Met, Arg, Val, Phe, Ser, Thr, Tyr, and Trp. This module contained 8 DE metabolites, the majority of which were decreased in CKD. In contrast, subnetwork 2 (9 nodes, 10 edges) represented the most coherent CKD-associated module, with all 9 metabolites differentially expressed. It included ASA and MetHis, together with short-chain, dicarboxylic, and hydroxylated acylcarnitines (C4DC, C5:1, C5OH, C5DC, C6DC, C8DC, and C8:1), all of which were increased in CKD. Subnetwork 3 (6 nodes, 5 edges) consisted of short- and medium-chain acylcarnitines (C2, C4-OH, C6, C8, C10:1, and C10), predominantly increased in CKD. The largest module was represented by subnetwork 4 (15 nodes, 19 edges) comprising medium- and long-chain acylcarnitines (e.g., C3DC, C12:1, C14:1, C14-OH, C16:1, C16:1-OH, and C18:1). This subnetwork contained 14 DE metabolites, most of which were increased in CKD.

#### 3.4.2. Node-Level Topological Reorganization

Topological differences between CKD and control networks were primarily driven by changes in betweenness centrality and stress, while degree and closeness centrality showed comparatively limited variation (Table S11). MetHis, C3DC, C5DC, C16:1, C16:1-OH, C12:1, C14, and C14-OH showed concurrent increases in betweenness centrality and stress in CKD, consistent with a strengthened role within the network backbone and increased participation in high-traffic paths. Among these, MetHis, C16:1, and C16:1-OH act as prominent bridge nodes in the CKD network, with elevated traffic load showing the largest increases in betweenness centrality and stress. In contrast, Phe and Thr presented bridge-like roles under CKD conditions exhibiting betweenness centrality and stress in CKD but not in the control network. Conversely, C4DC, C6DC, C14:2, C18:2, and C10:1 showed coordinated decreases in both betweenness centrality and stress in CKD, indicating a reduced role as bridge nodes and diminished participation in backbone network traffic. Closeness centrality remained largely stable across networks. However, Leu, Met, Tyr, and Val showed decreased closeness centrality in CKD. In addition, Arg, C3, and C5 lacked measurable closeness centrality in CKD while retaining values in the control network, consistent with a shift toward peripheral positioning.

#### 3.4.3. Integrated Changes in Network Edges

Edge-level differences between CKD and control networks (Table S12) revealed a combination of edge loss, edge gain, and modulation of existing connections, as reflected by changes in partial correlations (Δpcor). Loss of connectivity (edges present in the CTR network but absent in CKD) was observed for Leu–Met, Met–Arg, C3–C5, and C14:2–C18:2. In addition, several edges showed marked decreases in partial correlation, with the largest reductions observed for Leu–Phe (Δpcor ≈ −0.30) and Met–Phe (Δpcor ≈ −0.13), both of which remained present in CKD but weakened. Conversely, new connections emerged exclusively in the CKD network, including C5DC–MetHis, C12:1–C16:1, C14–C16:1-OH, C16:1-OH–C18:1-OH, and Ser–Thr, indicating the formation of condition-specific associations. Furthermore, several edges present in both networks showed increased coupling between metabolites (increased partial correlation in CKD), particularly within acylcarnitine-related subnetworks, ASA–C8DC (Δpcor ≈ +0.13), C4DC–C5DC (+0.10), and C4DC–C6DC (+0.08). In contrast, weakening of existing connections was particularly evident among amino acid interactions, including Leu–Phe, Met–Phe, and Tyr–Trp (Δpcor ≈ −0.08).

#### 3.4.4. Cross-Compartment Layer Distribution of Metabolites

Metabolites displayed a structured distribution across compartments when evaluated using log_2_-transformed urine-to-plasma ratios (U/P) derived from paired samples. A distinct group of metabolites was enriched in plasma, including amino acids and urea cycle intermediates such as Leu, Arg, Phe, Met, Trp, and Cit (log_2_ U/P up to ~4.0) together with two unsaturated long-chain acylcarnitines (C18:1, C18:2). In contrast, a large group of metabolites was markedly enriched in urine, dominated by dicarboxylic and hydroxylated acylcarnitines, including ASA, C6DC, C5:1, C4DC, and C14DC (log_2_ U/P ranging from approximately −2 to −3.5) while a mixed plasma–urine distribution was observed for intermediates of mitochondrial β-oxidation medium-chain acylcarnitines (C2, C3, C5, C6, C8, C10).

Mapping metabolites onto a two-dimensional space defined by plasma log_2_ fold change (CKD vs. CTR) and log_2_(U/P) ratios (Figure 3B) demonstrated a clear separation of metabolite classes according to both disease-associated changes and compartmental distribution. Metabolites from subnetwork 2, including C6DC, C5DC, C4DC, ASA, and MethylHis, were tightly clustered in the lower-right quadrant, characterized by both increased abundance in CKD and strong enrichment in urine. Subnetwork 3 metabolites (e.g., C4OH, C2, C10:1) occupied an intermediate position, reflecting CKD-associated increases with more moderate compartmental shifts.

In contrast, amino acids from subnetwork 1 were predominantly located in the plasma-enriched region and showed heterogeneous behavior in CKD. Several amino acids were decreased in CKD while remaining plasma-retained (e.g., Leu, Arg, Phe, Met, Trp), whereas others exhibited weaker changes or partial redistribution toward urine (e.g., Tyr, Thr, Ser, Val). Notably, long-chain acylcarnitines (e.g., C18:1, C18:2) showed plasma enrichment despite modest increases in CKD, indicating distinct handling compared to shorter-chain species.

#### 3.4.5. Urine Network Topology and Cross-Compartment Role Reorganization

**Figure 4:**
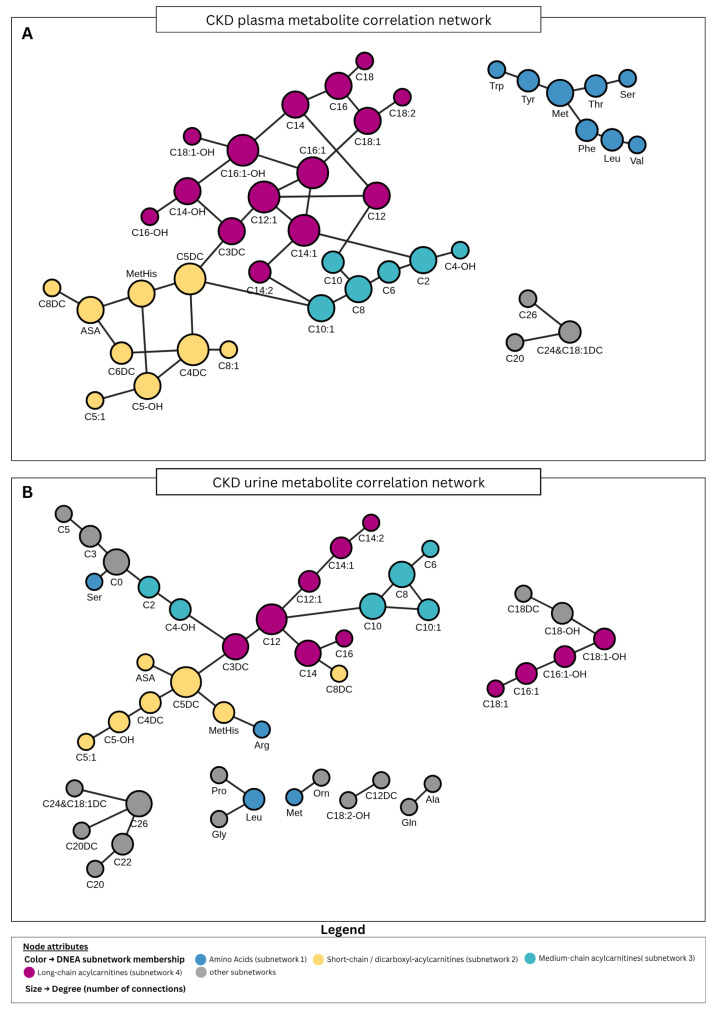
Network organization of metabolites in plasma and urine CKD. (**A**) CKD plasma metabolite correlation network showing metabolites modules. Node color reflects subnetwork membership, and node size is proportional to degree. (**B**) CKD urine metabolite correlation network. Node color reflects DNEA-derived subnetwork membership as defined in the plasma network, enabling direct comparison of metabolite roles between compartments. Node size is proportional to degree.

To further assess whether compartmental distribution was associated with changes in network organization, topological properties of the CKD urine network were examined (Figure 4A,B; Table S11). C5DC, C3DC, C12, C16:1-OH, and C18:1-OH metabolites that exhibited elevated betweenness centrality and stress in the plasma CKD network retained central positions in the CKD urine network. A subset of metabolites displayed compartment-specific centrality, emerging as prominent bridge nodes in the urine network despite more modest roles in plasma (C0, C2, and C4-OH, C26). Conversely, several metabolites (notably, C16:1 and C14-OH) that exhibited bridge or backbone characteristics in the plasma network showed reduced centrality in urine. Also, C4DC and C6DC remained weakly connected in urine. Amino acids, including Gly, Pro, Ser, Arg, and Met, were characterized by low degree and minimal betweenness centrality in the urine network, consistent with peripheral positioning.

## 4. Discussion

Profiling of AAs and ACs represents a well-established metabolomics approach applied extensively in newborn screening for the detection of inborn errors of metabolism and increasingly in cardiovascular disorders [24,25,26,27,28], diabetes [29,30,31], and CKD [32,33,34,35]. Given the central role of the kidney in amino acid handling, nitrogen balance, and fatty acid oxidation [36,37], combined analysis of plasma and urine AAs and ACs provides a valuable framework for exploring both systemic and urinary metabolic alterations associated with CKD. In this context, this study investigated CKD-associated metabolic alterations in AAs and ACs metabolism of 148 participants, comprising 78 patients with CKD and 70 controls with preserved renal function, using an integrated analytical framework combining covariate-adjusted differential analysis, supervised multivariate modeling, DNEA, and cross-compartment plasma–urine profiling. The CKD cohort covered a broad spectrum of disease severity, predominantly stages 3 and 4, and was characterized by reduced eGFR and a high prevalence of hypertension and diabetes mellitus, comorbidities that are themselves known to influence amino acid and acylcarnitine metabolism. Nevertheless, when these clinical variables were integrated into the correlation network analysis alongside the measured metabolites, neither diabetes nor hypertension emerged as dominant hubs within the network topology. This observation suggests that, despite their substantial contribution to the overall comorbidity burden, these conditions do not primarily drive the metabolic alterations captured by the present analytical approach. At the same time, it should be noted that the high prevalence of both comorbidities is an inherent characteristic of advanced CKD populations, and their potential confounding effect cannot be entirely eliminated. While age and sex were included as covariates in all differential analyses, the potential residual influence of diabetes mellitus, hypertension, and associated medications on the observed metabolic profiles cannot be fully excluded. The identified signatures should therefore be interpreted as CKD-associated metabolic alterations rather than CKD-specific findings, pending validation in comorbidity-stratified cohorts. Importantly, both plasma and urine samples were available for CKD patients, whereas only plasma samples were collected from controls. This design enabled the integration of disease-associated metabolic alterations (CKD vs. CTR in plasma) with compartment-specific analyses within CKD patients (plasma vs. urine), providing complementary insights into systemic and renal aspects of metabolic reprogramming while also constituting a limitation for cross-compartment interpretation, as discussed below.

### 4.1. Networks—Correlation Networks and DNEA

The clinical correlation network was organized into three interconnected domains: renal function (CKD stage, eGFR, creatinine, urea), mineral–bone metabolism (phosphate, calcium, PTH), and hematological parameters (hemoglobin, hematocrit). While the central role of renal function is expected in CKD, the observed network structure emphasizes strong integration with mineral–bone and hematological domains, with phosphate acting as a key connector, consistent with its recognized role in linking renal dysfunction to systemic metabolic disturbances [38]. In contrast, metabolic, inflammatory, and cardiovascular variables showed weaker associations and remained peripheral, suggesting a more limited contribution to the core clinical network in this cohort.

In plasma, kidney function markers were strongly associated with Cit, ASA, MetHis, and Trp, forming a coherent metabolic module. These metabolites are functionally linked through nitrogen metabolism, encompassing urea cycle intermediates (Cit, ASA), markers of protein turnover (MetHis), and amino acid-derived pathways (Trp). Their coordinated association with renal function is consistent with CKD-associated alterations in renal amino acid and nitrogen metabolism reported previously [10,39,40,41,42,43,44]. In parallel, dicarboxylic and hydroxylated acylcarnitines (C3DC, C4DC, C5DC, C6DC, C8DC, C5:1, and C5-OH) displayed strong associations with kidney function markers and high network connectivity, consistent with previous metabolomics studies linking reduced kidney function to alterations in acylcarnitines metabolism [42,45,46].

Several amino acids showed distinct associations with kidney function parameters across plasma and urine. In plasma, Glu was linked to CKD stage, eGFR, and creatinine, while Asp was primarily associated with CKD stage. In urine, amino acids displayed more heterogeneous patterns, with Val, Lys, and Pro associated with filtration markers, whereas His and Ser were linked to urea. Overall, amino acid metabolites appear to reflect distinct aspects of renal dysfunction depending on the specific kidney function parameter considered. Mineral–bone metabolism, primarily regulated through endocrine pathways involving phosphate, PTH, and vitamin D, showed limited overlap with the metabolite network, with only a few plasma metabolites (ASA, MetHis, C4DC) and urinary Cit involved. The hematological axis showed broader, integrated associations with metabolites, particularly in plasma. Hemoglobin and hematocrit were linked to a wide range of metabolites, including AAs and both short- and long-chain ACs, indicating that CKD-related hematological changes are embedded in systemic metabolism. This aligns with the multifactorial nature of CKD-associated anemia, which involves impaired erythropoietin production, inflammation, and metabolic stress [47]. In urine, these associations were more limited, supporting the view that hematological alterations are primarily reflected at the systemic level rather than through renal excretion patterns.

DNEA was applied to move beyond pairwise correlations and capture higher-order changes in metabolic organization, enabling the identification of coordinated condition-specific alterations in metabolite networks by integrating differential abundance with network topology. This framework was used to assess: (i) disease-associated changes in plasma networks comparing CKD and CTR groups, and (ii) compartment-specific organization assessed through comparison of plasma and urine networks within CKD patients. In the plasma CKD vs. CTR comparison, DNEA identified six subnetworks, of which four remained significant after FDR correction. Significant subnetworks mapped to distinct metabolite classes and were enriched in differentially abundant metabolites, indicating that CKD-related changes form coherent metabolic modules. A clear separation emerged: the AA-enriched module was predominantly composed of metabolites decreased in CKD, whereas AC-dominated modules were increased. This opposing behavior highlights two distinct metabolic axes in CKD plasma, reflecting reduced integration of amino acid metabolism alongside coordinated accumulation of acylcarnitines. These patterns were reflected in network topology, with increased metabolites (notably acylcarnitines and MetHis) gaining centrality, while several amino acids became more peripheral. At the metabolite–metabolite interaction level, these shifts were accompanied by selective network rewiring with strengthened acylcarnitine connections and weakened amino acid interactions in CKD.

Together, these findings show that acylcarnitine pathways become more connected and central in CKD, while amino acid metabolism loses integration—a systemic reorganization that extends, albeit with compartment-specific modifications, into the urinary network. Notably, the observed differences in metabolite centrality between plasma and urine networks were identified within CKD patients only, as urine samples from healthy controls were not available; the plasma–urine comparisons therefore characterize compartment-specific metabolic organization in disease rather than deviation from a healthy urinary reference, a distinction that should be considered when interpreting these findings.

### 4.2. Differential Metabolite Analysis, Pathway Interpretation, and Compartmental Distribution

Exploratory analysis showed that metabolic differences were driven by CKD status and were not influenced by demographic variables, supporting the robustness of the differential metabolite results. The observed CKD metabolic alterations are dominated by two opposing trends accumulation of acylcarnitines, a pattern consistent with incomplete fatty acid oxidation, and depletion of several amino acids, suggestive of altered protein metabolism and/or increased utilization. Given the observational nature of this study and the high prevalence of comorbidities in the cohort, the following pathway interpretations should be understood as biologically plausible associations supported by the prior literature and not as direct mechanistic evidence.

#### 4.2.1. Acylcarnitine Accumulation and Mitochondrial Fatty-Acid Oxidation

Short-chain dicarboxylic acylcarnitines originate from amino acid and tricarboxylic acid cycle metabolism and from peroxisomal shortening of longer dicarboxylic species, whereas medium-chain forms may arise during incomplete β-oxidation with compensatory ω-oxidation [48]. Their elevation (C3DC, C4DC, C5DC, C6DC) in CKD patients’ plasma and urine is consistent with altered fatty-acid oxidation, a process previously associated with tubular dysfunction in CKD. Importantly, free carnitine levels were not altered in this cohort, consistent with reports indicating that carnitine depletion primarily occurs after the initiation of hemodialysis [49,50] and supporting the view that the observed acylcarnitine accumulation reflects metabolic rather than carnitine-supply-related alterations.

#### 4.2.2. Elevated Methylhistidine and Argininosuccinic Acid: Markers of Protein Catabolism and Impaired Renal Nitrogen Metabolism

Elevated plasma MetHis levels enhanced myofibrillar protein catabolism [10,51], a pattern supported by the concurrently high urinary MetHis excretion observed in this cohort. Of the two isoforms, only 3-MetHis reflects net protein breakdown, since it originates from the post-translational methylation of histidine residues of actin and myosin and is released during myofibrillar protein degradation [52], while 1-MetHis is primarily derived from dietary sources (e.g., anserine) [53]. Still, since isoform-specific quantification was not performed in this study, the contribution of myofibrillar protein catabolism to the observed MetHis elevation cannot be directly determined. Similarly to MetHis, CKD patients presented elevated plasma of ASA (↑ urine excretion), one of the key metabolites in urea cycle. This pathway is normally active in proximal tubular cells [40], where ASA is produced by argininosuccinate synthase from Cit, then subsequently metabolized to Arg by argininosuccinate lyase, making the kidney a key contributor to systemic Arg homeostasis [43,44]. Loss of functional tubular mass and metabolic capacity, together with reduced renal clearance, may contribute to ASA accumulation in CKD although the relative contribution of impaired enzymatic activity versus reduced clearance cannot be determined from the current data.

#### 4.2.3. Amino Acid Depletion: Aromatic Amino Acids and One-Carbon Metabolism

In CKD, alterations in aromatic amino acids were evident, being characterized by significantly decreased Trp plasma levels (↓ urine excretion), as well as reduced Tyr plasma levels (↑ urine excretion), accompanied by increased plasma Phe/Tyr ratio. This pattern aligns with reports of overall lower circulating aromatic amino acids in CKD [54]—a pattern consistent with inflammation-driven activation of indoleamine-2,3-dioxygenase and diversion of Trp through the kynurenine pathway [55], which has been reported to correlate with disease severity [56]. In parallel, the decreased Tyr levels together with the increased Phe/Tyr ratio are consistent with impaired renal conversion of Phe to Tyr, a process known to decline in CKD, particularly in advanced stages [42,57]. Although these findings indicate altered aromatic amino acid metabolism, their clinical implications remain incompletely defined.

Lower Ser (↑ urine excretion) and Met (↓ urine excretion) levels may suggest alterations in one-carbon metabolism. As the kidney is a major source of circulating Ser, its depletion in CKD is consistent with reduced renal biosynthetic capacity, with potential consequences for homocysteine remethylation, methylation reactions, and sphingolipid synthesis, usually implicated in uremic neuropathy [58,59,60,61]. Lower Met may reflect impaired S-adenosylmethionine–dependent methylation and has been associated with rapid CKD progression independent of baseline eGFR [62].

Plasma Val levels were significantly decreased in CKD compared to CTR, accompanied by increased urinary excretion. Previous reports indicate that Val is a candidate biomarker to distinguish between diabetic kidney disease and type 2 diabetes [63], while decreased levels of branched-chain amino acids are expected in CKD patients due to reduced intake and increased muscle oxidation [64]. As a key substrate in branched-chain amino acid metabolism, Val undergoes mitochondrial transamination followed by oxidative decarboxylation to form isobutyryl-CoA, linking it to downstream pathways such as odd-chain fatty acid synthesis and further to the generation of short-chain acylcarnitines [65].

#### 4.2.4. eGFR-Dependent Versus eGFR-Independent Metabolic Alterations

A large fraction of the changes observed through uni- and multivariate approaches are driven by renal function, considering that after eGFR correction, 68 metabolites lose their significance (especially short-chain ACs, dicarboxylic and hydroxylated ACs, MetHis, ASA), indicating their close link to reduced renal clearance and/or filtration-dependent accumulation. Still, 12 metabolites, representative of AAs (Tyr, Met, Arg) and medium- and long-chain ACs, retain their significance. This distinction is directly relevant to biomarker interpretation: candidates drawn from the eGFR-attenuated set primarily reflect filtration-dependent accumulation, whereas the eGFR-robust subset (C6, C8, C10; Tyr, Met, Arg) is more likely to capture intrinsic metabolic reprogramming. Of these, the three medium-chain ACs also displayed a FC > 1.2 after eGFR correction; these metabolites have been previously documented as increased in diabetes [47]. Despite the high prevalence of diabetes in the studied cohort (53.8%), no significant correlation was identified between these acylcarnitines and diabetes status in the current dataset, suggesting that their elevation may be related to CKD-associated metabolic alterations rather than exclusively to glycemic dysregulation. However, this observation should be interpreted cautiously given the limited power to detect diabetes-specific effects without a matched non-diabetic CKD subgroup. Dedicated studies with appropriate comorbidity-stratified designs are needed to confirm the CKD-specificity of these alterations.

#### 4.2.5. Multivariate Confirmation and Convergent Feature Selection

Supervised multivariate analysis by PLS-DA provided complementary support for the pathway-level alterations identified through univariate approaches, with the first two components explaining approximately 40% of total variance (Figure S3). Given that PLS-DA is a supervised method, these findings should be interpreted as exploratory, particularly in the context of moderate sample size of the present cohort. VIP-selected metabolites (>1.5) overlapped with those identified in covariate-adjusted univariate models, supporting the robustness of feature selection and, indicating consistency between analytical approaches highlighting a limited set of metabolites. Besides MetHis, ASA, Trp and dicarboxylic acylcarnitines C3DC, C4DC, C5DC, C6DC, other features—namely, MetHis Synthesis (MetHis to His ratio), NOS activity (Cit to Arg ratio), C5:1, C5-OH, C14-OH and C5DC to C16 ratio—also contributed substantially to group separation, emphasizing their involvement in CKD-related metabolic dysregulation. Overall, convergence between the univariate, supervised multivariate, and network-based analyses provides complementary support for the identified CKD-associated metabolic signature.

#### 4.2.6. Multi-Marker Biomarker Panel

Univariate biomarker analysis identified several metabolites with strong discriminatory performance (AUC > 0.85) for CKD vs. control, concentrated within the metabolic clusters most enriched in differentially abundant metabolites. A multi-marker panel was constructed using a cluster-based approach selecting one representative from each of three distinct metabolic clusters: MetHis (Cluster 4; protein turnover/nitrogen metabolism), C3DC (Cluster 5; acylcarnitines/fatty-acid oxidation), and Trp (Cluster 3; aromatic amino acid/kynurenine metabolism). This approach aimed to capture complementary metabolic alterations while minimizing redundancy among correlated metabolites. The three-metabolite panel achieved an AUC of 0.881 (95% CI 0.813–0.938), with overall accuracy of 0.851, sensitivity of 0.824, and specificity of 0.878, indicating good discriminatory performance within the present cohort and reducing both false negatives (17 to 13) and false positives (10 to 9) (Figure 2). These results should be interpreted as exploratory, being only discovery-phase estimates. The panel was evaluated on the same dataset used for feature selection, without an independent external validation cohort, and the reported AUC values may therefore be optimistic, although repeated cross-validation and permutation testing (*n* = 1000) were used to reduce the risk of overfitting, but do not substitute for external validation. Compared to previous studies, other MetHis-containing biomarker panels offered similar sensitivity, but higher accuracy [66]. Nevertheless, this clustering-guided strategy reduces redundancy and captures complementary metabolic information, supporting a pathway-based approach. It is also important to note that the proposed panel is not intended as a replacement for established diagnostics such as serum creatinine and eGFR, which remain the clinical standard for CKD diagnosis. Rather, the proposed panel should be considered an exploratory candidate metabolic signature that may provide complementary information on CKD-associated metabolic alterations. If validated in independent and clinically well-characterized cohorts, it may contribute to metabolic phenotyping and patient stratification alongside established clinical biomarkers.

#### 4.2.7. Limitations, Strengths of the Study and Future Directions

Several limitations of this study need to be acknowledged. One of the main limitations is that the observational cross-sectional design precludes causal inference; all pathway interpretations should be considered biologically plausible associations rather than established mechanistic conclusions. Although the study included patients across CKD stages 2–5, the unequal distribution of participants among disease stages, particularly the limited number of stage 2 and stage 5 patients, prohibited statistically robust stage-specific subgroup analyses. Moreover, considering that the control plasma samples were sourced from a parallel study, and as such, clinical metadata beyond age, sex, and eGFR were not available, it may limit the extent to which potential confounding factors can be accounted for in the statistical comparisons, warranting cautious interpretation of the observed differences. Similarly, the absence of urine from healthy controls means that cross-compartment analyses reflect metabolite distribution patterns within disease rather than deviation from a healthy urinary baseline, limiting the interpretation of compartment-specific findings.

While age and sex were included as covariates, residual confounding from diabetes mellitus and hypertension cannot be excluded, even though correlation analysis did not highlight them as relevant hubs in network topology. The same is true for the associated pharmacological treatments—including renin–angiotensin–aldosterone system inhibitors, statins, and antidiabetic agents, which might influence amino acid and acylcarnitine profiles. The identified metabolic signatures should therefore be interpreted as CKD-associated rather than CKD-specific until validated in better-characterized, comorbidity-stratified cohorts. Finally, the candidate biomarker panel was derived and evaluated within the same dataset without an external validation cohort, and the reported performance metrics should be considered exploratory. Also, the analytical characteristics of FIA-MS/MS should be considered when interpreting the findings. Because FIA-MS/MS does not involve chromatographic separation, compounds with identical or overlapping mass transitions, including isobaric and structural isomers (such as 2-MetHis and 3-MetHis), are not individually resolved. Moreover, the simultaneous introduction of sample components into the ion source may increase susceptibility to matrix-related ion suppression or enhancement, even though these effects should be largely accounted for by the isotope-labeled internal standards.

These limitations define clear priorities for future work. Studies with larger, prospectively recruited cohorts that include comorbidity-stratified subgroups will be essential to confirm the CKD-specificity of the identified metabolic signature and to evaluate the independent contribution of diabetes and hypertension. Longitudinal designs are needed to determine whether the identified metabolites have prognostic value across CKD progression stages. Expanding the targeted panel to include downstream kynurenine pathway metabolites would allow more direct testing of the Trp- indoleamine-2,3-dioxygenase hypothesis proposed here. Moreover, isoform-specific quantification of MetHis would strengthen the protein catabolism interpretation. The inclusion of matched urine samples from healthy controls in future studies would substantially improve cross-compartment analysis and support a more rigorous evaluation of renal handling and metabolite redistribution. The strengths of this study include the application of an integrated multi-level analytical framework—combining covariate-adjusted linear models, supervised multivariate analysis, DNEA, and cross-compartment profiling—which provides convergent and mutually reinforcing evidence for the identified metabolic signature. The concordance of findings across independent analytical approaches (limma, PLS-DA, DNEA, and correlation network analysis) reduces the likelihood that the reported associations are analytical artifacts. The dual-compartment design, while limited by the absence of control urine, enabled a distinction between systemic and potentially urinary metabolic alterations that single-compartment studies cannot provide. Finally, the clustering-guided biomarker panel construction strategy—selecting representatives from distinct metabolic modules rather than top-ranked individual markers—captures complementary biological information and reduces redundancy, offering a methodologically transparent approach to candidate panel development.

## 5. Conclusions

In conclusion, targeted plasma metabolomics identified a CKD-associated profile characterized by elevated short-chain dicarboxylic acylcarnitines together with increased MetHis and ASA and reduced Trp, Ser, Met, and Tyr. This pattern is consistent with alterations in fatty-acid oxidation, protein turnover and nitrogen metabolism, renal Arg synthesis, one-carbon and aromatic amino acid metabolism. The consistency of these metabolites across statistical analyses, together with their correlation with kidney function markers, supports their biological relevance and potential value for further investigation as candidate biomarkers. However, many of the observed metabolic alterations were attenuated after adjustment for eGFR, highlighting the substantial influence of kidney function on the identified CKD-associated profile. Future longitudinal studies in larger, balanced cohorts across CKD stages are planned to validate the proposed biomarker panel and evaluate its diagnostic and prognostic utility and potential clinical relevance in CKD-stage differentiation.

## Figures and Tables

**Table 1:** Demographic and clinicopathological characteristics of patients and controls.

	CKD (*n* = 78)	CTR (*n* = 70)	*p*-Value
**Characteristic**			
Age (years)	67 ± 12.4	66 ± 11.5	0.50
Male gender (%)	41 (52.6%)	48 (68.6%)	0.05
Body mass index (BMI, kg/m^2^)	28.5 (25.7–33.2)	-	-
eGFR (mL/min/1.73 m^2^)	32.6 ± 16.6	92.9 ± 16.0	-
Serum creatinine (mg/dL)	1.8 (1.4–2.6)	0.8 (0.7–1.0)	-
**CKD stage, *n* (%)**			
Stage 2	5 (6.4%)	-	-
Stage 3	38 (48.7%)	-	-
Stage 4	24 (30.8%)	-	-
Stage 5	11 (14.1%)	-	-
**Comorbidities**			
Diabetes mellitus, *n* (%)	42 (53.8%)	-	-
Hypertension, *n* (%)	74 (94.9%)	-	-

Note: Continuous variables are presented as mean ± SD or median (interquartile range, Q1–Q3) and categorical variables are presented as number (percentage).

## Data Availability

Data are provided within the Supplementary Information Files.

## Supplementary Materials

The following supporting information can be downloaded at: https://www.mdpi.com/article/10.3390/biomedicines14071622/s1, Figure S1: Network representation of significant correlations. Network representation of significant Kendall’s tau correlations (*p* < 0.05, |τ| > 0.26) among clinical variables (A), plasma metabolites and clinical variables (B), and urine metabolites and clinical variables (C) in CKD patients. Nodes are color-coded according to variable class (clinical domains or metabolite classes, see legend) and node size is proportional to node degree (number of significant connections). Edges represent pairwise correlations, with red indicating positive and blue indicating negative associations; edge thickness is proportional to correlation strength (|τ|). Only statistically significant correlations exceeding the moderate Kendall threshold (|τ| > 0.26) are displayed. Figure S2: Exploratory analysis of metadata and global metabolomic patterns. (A) Correlation heatmap of sample metadata. Kendall’s rank correlation coefficients (τ) were calculated to assess associations among age, sex, and group classification (CKD vs. CTR). Color intensity reflects the strength and direction of the correlations; (B) PCA scores plots of plasma metabolomic profiles. (C) Hierarchical clustering heatmap of plasma metabolomic profiles. Sample annotation bars indicate group, age, and sex. Figure S3: Supervised multivariate analysis of metabolomic profiles using PLS-DA. Left panel: Pairwise score plots of the first five PLS-DA components. The diagonal panels indicate the percentage of variance explained by each component; Upper right panel: Score plot of component 1 versus component 2, demonstrating group separation between CKD and CTR samples. Shaded ellipses represent 95% confidence intervals for each group; Lower right panel: Model performance and validation metrics. Bar plot shows classification accuracy, R^2^, and Q^2^ values across models including 1–5 components. Permutation testing (2000 permutations) demonstrates that the observed model performance exceeds that expected by chance (empirical *p* < 5 × 10^−4^). Figure S4: Representative receiver operating characteristic (ROC) curves. Metabolites were selected to illustrate cluster composition and general discriminatory performance across clusters. ROC curves are shown with 95% confidence intervals. Table S1: Other demographic and clinicopathological characteristics of patients; Table S2: List of metabolites, metabolite sums and ratios; Table S3. Metabolites MS/MS acquisition parameters; Table S4: Plasma metabolites measurements in CKD patients and controls; Table S5: Urine ratios of metabolites to creatinine concentrations in CKD patients; Table S6: Correlation analysis of clinical variables with metabolites determined in urine and plasma of CKD patients; Table S7: Network topology parameters for correlation networks for plasma and urine of CKD patients; Table S8: Limma linear modeling results for differential plasma metabolites between CKD and CTR groups, with and without adjustment for age and sex covariates; Table S9: VIP scores for metabolites across PLS-DA components in CKD vs. CTR; Table S10: Performance metrics from classical univariate ROC curve analysis of individual metabolites distinguishing CKD from CTR; Table S11: DNEA plasma subnetworks and topology (CKD vs. CTR), plasma–urine node-level and cross-compartment distribution in CKD; Table S12: Partial correlation changes for metabolite–metabolite edges in CKD and CTR plasma networks.
