# Supplementary material for: FIA-MS/MS-Based Targeted Metabolomics of Amino Acids and Acylcarnitines Uncovers Network-Level Metabolic Reprogramming in Chronic Kidney Disease
Source: Biomedicines. 2026 Jul 18;14(7):1622. doi: 10.3390/biomedicines14071622 (PMC13406151; doi:10.3390/biomedicines14071622)
Supplement: Supplementary file 1 [file biomedicines-14-01622-s001.zip › biomedicines-4389711 supplementary Figures.pdf]

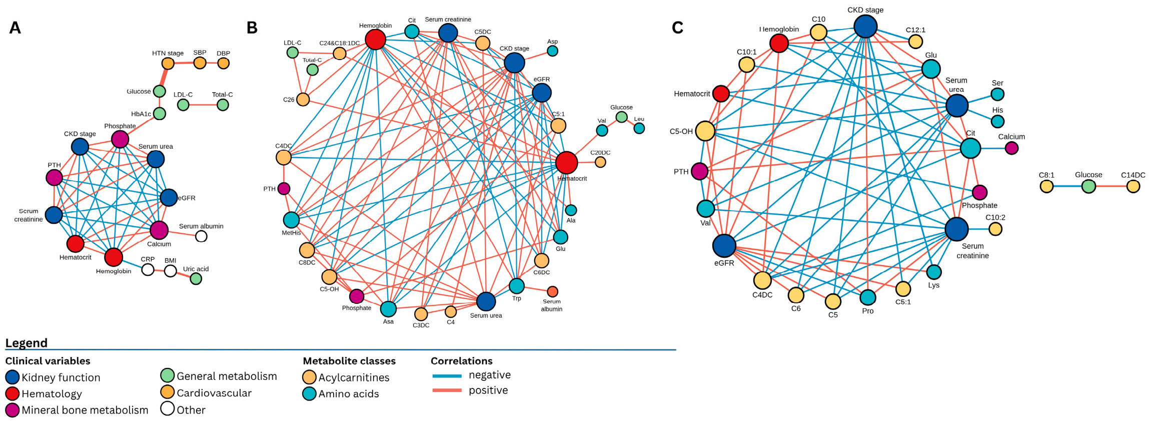

Figure S1. Network representation of significant correlations. Network representation of significant Kendall's tau correlations ($p < 0.05$, $|\tau| > 0.26$) among clinical variables (A), plasma metabolites and clinical variables (B), and urine metabolites and clinical variables (C) in CKD patients. Nodes are color-coded according to variable class (clinical domains or metabolite classes, see legend) and node size is proportional to node degree (number of significant connections). Edges represent pairwise correlations, with red indicating positive and blue indicating negative associations; edge thickness is proportional to correlation strength ($|\tau|$). Only statistically significant correlations exceeding the moderate Kendall threshold ($|\tau| > 0.26$) are displayed.

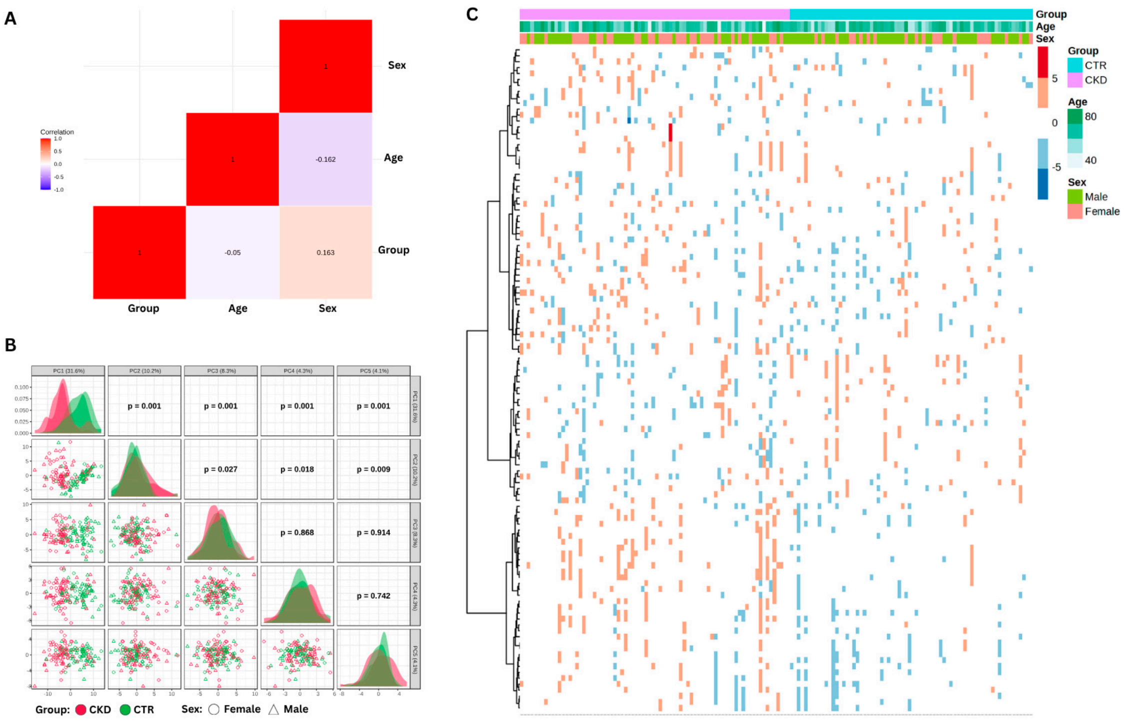

Figure S2. Exploratory analysis of metadata and global metabolomic patterns. (A) Correlation heatmap of sample metadata. Kendall's rank correlation coefficients (τ) were calculated to assess associations among age, sex, and group classification (CKD vs. CTR). Color intensity reflects the strength and direction of the correlations; (B) PCA scores plots of plasma metabolomic profiles. (C) Hierarchical clustering heatmap of plasma metabolomic profiles. Sample annotation bars indicate group, age, and sex.

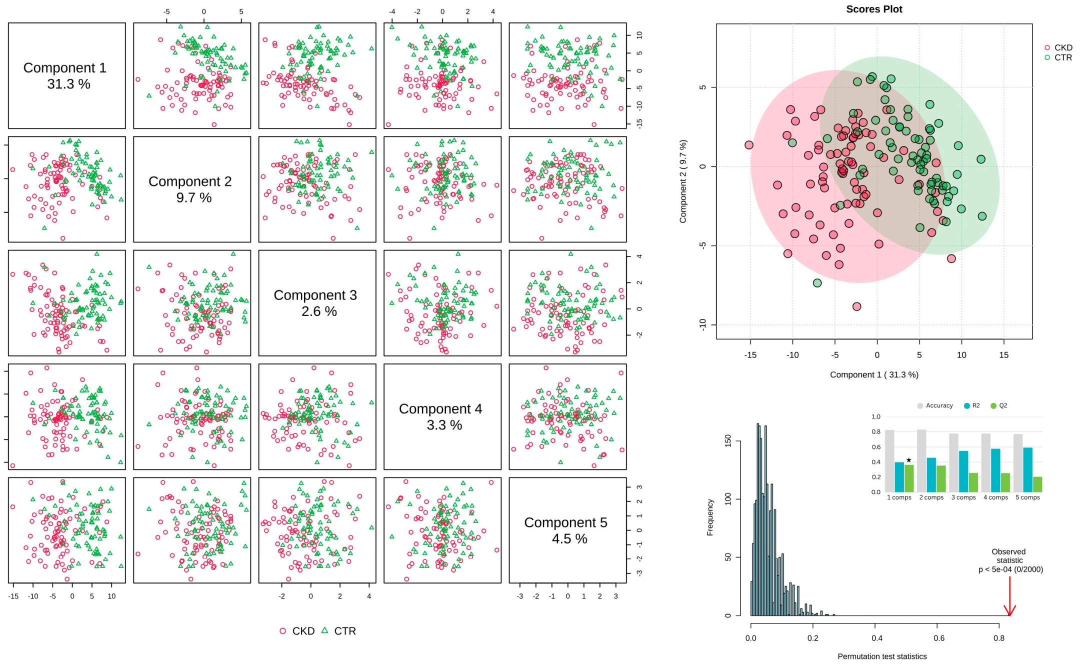

Figure S3. Supervised multivariate analysis of metabolomic profiles using PLS-DA. Left panel: Pairwise score plots of the first five PLS-DA components. The diagonal panels indicate the percentage of variance explained by each component; Upper right panel: Score plot of component 1 versus component 2, demonstrating group separation between CKD and CTR samples. Shaded ellipses represent 95% confidence intervals for each group; Lower right panel: Model performance and validation metrics. Bar plot shows classification accuracy, R^2, and Q^2 values across models including 1–5 components. Permutation testing (2000 permutations) demonstrates that the observed model performance exceeds that expected by chance (empirical $p < 5 \times 10^{-4}$).

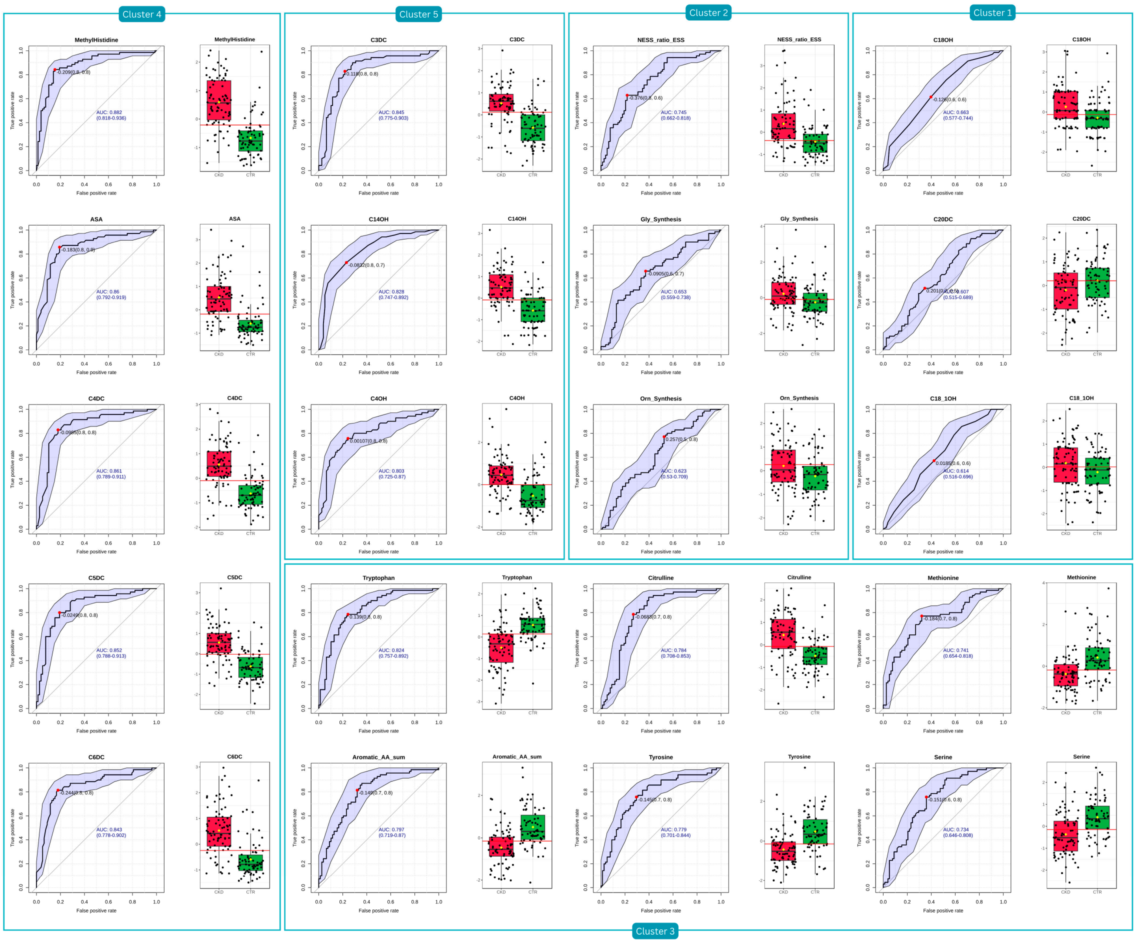

Figure S4. Representative receiver operating characteristic (ROC) curves. Metabolites were selected to illustrate cluster composition and general discriminatory performance across clusters. ROC curves are shown with 95% confidence intervals.
